# Closing the computational biology ‘knowledge gap’: Spanish Wikipedia as a case study

**DOI:** 10.1093/bioinformatics/btae247

**Published:** 2024-06-28

**Authors:** Nelly Sélem-Mojica, Tülay Karakulak, Audra Anjum, Antón Pashkov, Rafael Pérez-Estrada, Karina Enriquez-Guillén, Dan DeBlasio, Sofia Ferreira-Gonzalez, Alejandra Medina-Rivera, Daniel Rodrigo-Torres, Alastair M Kilpatrick, Lonnie R Welch, Farzana Rahman

**Affiliations:** Centro de Ciencias Matemáticas, Universidad Nacional Autónoma de México, Morelia, 58089, Mexico; Department of Molecular Life Sciences and Swiss Institute of Bioinformatics, University of Zurich, Zurich, 8057, Switzerland; Department of Pathology and Molecular Pathology, University Hospital Zurich, Zurich, 8091, Switzerland; Office of Instructional Design, Ohio University, Athens, OH, 45701, United States; Escuela Nacional de Estudios Superiores (ENES) Unidad Morelia, Universidad Nacional Autónoma de México, Morelia, 58190, Mexico; Escuela Nacional de Estudios Superiores (ENES) Unidad Morelia, Universidad Nacional Autónoma de México, Morelia, 58190, Mexico; Escuela Nacional de Estudios Superiores (ENES) Unidad Morelia, Universidad Nacional Autónoma de México, Morelia, 58190, Mexico; Ray and Stephanie Lane Computational Biology Department, Carnegie Mellon University, Pittsburgh, PA, 15213, United States; Centre for Regenerative Medicine, Institute for Regeneration and Repair, The University of Edinburgh, Edinburgh, EH16 4UU, United Kingdom; Centre for Inflammation Research, Institute for Regeneration and Repair, The University of Edinburgh, Edinburgh, EH16 4UU, United Kingdom; Laboratorio Internacional de Investigación sobre el Genoma Humano, Universidad Nacional Autónoma de México, Juriquilla, 76230, Mexico; Centre for Regenerative Medicine, Institute for Regeneration and Repair, The University of Edinburgh, Edinburgh, EH16 4UU, United Kingdom; Centre for Regenerative Medicine, Institute for Regeneration and Repair, The University of Edinburgh, Edinburgh, EH16 4UU, United Kingdom; School of Electrical Engineering and Computer Science, Ohio University, Athens, OH, 45701, United States; School of Computer Science and Mathematics, Faculty of Engineering, Computing and the Environment, Kingston University, London, KT1 2EE, United Kingdom

## Abstract

**Motivation:**

Wikipedia is a vital open educational resource in computational biology. The quality of computational biology coverage in English-language Wikipedia has improved steadily in recent years. However, there is an increasingly large ‘knowledge gap’ between computational biology resources in English-language Wikipedia, and Wikipedias in non-English languages. Reducing this knowledge gap by providing educational resources in non-English languages would reduce language barriers which disadvantage non-native English speaking learners across multiple dimensions in computational biology.

**Results:**

Here, we provide a comprehensive assessment of computational biology coverage in Spanish-language Wikipedia, the second most accessed Wikipedia worldwide. Using Spanish-language Wikipedia as a case study, we generate quantitative and qualitative data before and after a targeted educational event, specifically, a Spanish-focused student editing competition. Our data demonstrates how such events and activities can narrow the knowledge gap between English and non-English educational resources, by improving existing articles and creating new articles. Finally, based on our analysis, we suggest ways to prioritize future initiatives to improve open educational resources in other languages.

**Availability and Implementation:**

Scripts for data analysis are available at: https://github.com/ISCBWikiTeam/spanish.

## 1 Introduction

Wikipedia is an online encyclopaedia and open education resource (OER), offering no-cost access, use, adaptation, and redistribution with no or limited restrictions (Miao *et al.* 2016). OERs are steadily becoming more popular amongst educators, as learners continue to find OERs to be as useful as traditional course materials (Abramovich and McBride 2018). Wikipedia is one of the most frequently visited websites in the world and is the most widely accessed educational resource in the field of computational biology (Kilpatrick *et al.* 2022). For instance, the English language article on Bioinformatics was viewed more than 270 000 times in 2023. The English-language Wikipedia (henceforth ‘English Wikipedia’) is the largest Wikipedia operated by the non-profit Wikimedia Foundation, with over 6.79 million articles and 47.07 million users as of March 2024. The Wikimedia Foundation operates an additional 325 Wikipedias in non-English languages, with 17 of these having more than 1 million articles.

Expert computational biologists have contributed content to English Wikipedia since at least 2007, when the Computational Biology taskforce of WikiProject Molecular Biology (formerly WikiProject Computational Biology) was founded. The taskforce aims to organize and improve Wikipedia articles relating to computational biology, of which there are now more than 1500 (O’Neill *et al.* 2017, Kilpatrick *et al.* 2022). Currently, about 47% of English Wikipedia computational biology articles still lack corresponding articles in any other language (Kilpatrick *et al.* 2022), demonstrating a clear ‘knowledge gap’ between computational biology content on English Wikipedia and Wikipedias in other languages. This disparity may be a result of promoting English language as a scientific *lingua franca*, of sorts, within certain databases of indexed journals (O’Neil 2018). Also, the current number of non-native speakers of English are more than double the number of native speakers (Eberhard *et al.* 2023), creating an increased demand for English Wikipedia content, compared to other languages. However, there are well-documented incentives to closing this scientific knowledge gap through the generation of OERs and other reference work in non-English languages. Increasing the access of scientific literature to populations that do not speak English or may not have access to paywalled scientific literature can encourage participation in STEM fields (McDermott 2023), allow non-native speakers of English to explore domain-specific knowledge without the added burden of second language acquisition (Amano *et al.* 2023), generate more culturally and contextually relevant resources (Cobo 2013), and address biases, especially for scientific reference materials such as systematic reviews and meta-analyses (Amano *et al.* 2016, Angulo *et al.* 2021).

Entire fields of research and creative activity stand to gain significant growth and advancement by encouraging non-English work. Thus, production of non-English OERs offers additional benefits to the scientific community worldwide. Calls to diversify the scientific *lingua franca* entail top-down, multi-agency, large-scale translation efforts, engaging professional translation services to lessen the knowledge gap (Henry *et al.* 2021). While these efforts can potentially improve the accessibility of academic research, we also maintain the position that grassroots efforts among scientists to increase the generation of scientific reference materials (e.g. Wikipedia articles relating to computational biology) is a more immediate and attainable goal. To this end, mentored contributions by non-professional translators working in non-English languages on the Wikipedia platform present a plausible and economical pathway toward the aim of increasing culturally responsive and linguistically accurate computational biology OERs. While Wikipedia does not publish primary research, it is an important resource of synthesized and distributed domain knowledge. The platform’s infrastructure and inherent participatory culture can foster the rapid development of scientific corpora in languages other than English.

Underrepresented languages are indicative of underrepresented voices, and highlight communities less able to access and contribute to computational biology. However, language barriers present several challenges to non-native English speakers in science in multiple ways, including knowledge access, career development, writing and publishing manuscripts, and contributing to global scientific discourse (Ramírez-Castañeda 2020). Thus, it is plausible that the amount of time and efforts non-native English speakers spend on reading, writing papers, or proofreading their papers is higher than the time spent by native English speakers. Recent estimates of the number of Spanish speakers worldwide range between 559 and 591 million, with 460 million native speakers (Eberhard *et al.* 2023, Loureda Lamas *et al.* 2023). It is also estimated that 7.9% of internet users speak Spanish (https://www.internetworldstats.com/stats7.htm), making it the 3rd most common language on the internet, after English and Mandarin Chinese. While English is a second language for many Spanish speakers, there is a considerable population of monolingual Spanish speakers (estimated to be almost 46% of Spaniards aged 25–64 in 2016; https://www.voanews.com/a/europe_why-spaniards-arent-learning-english-fast-rest-europe/6198760.html), highlighting the importance of having educational resources in Spanish. The Spanish language Wikipedia (*Wikipedia en español*; henceforth ‘Spanish Wikipedia’) has the second-highest number of users after English (7.09 million, as of March 2024) and is the eighth largest Wikipedia by number of articles (1.94 million) (https://meta.wikimedia.org/wiki/List_of_Wikipedias). Spanish scientists spend almost 100 additional hours writing a scientific article in English, compared to Spanish (Ramírez-Castañeda 2020). Similarly in a recent study, 36% of early career researchers with low or moderate English proficiency reported they would ‘often’ or ‘always’ avoid oral presentations at conferences due to English barriers (Amano *et al.* 2023).

A recent science communication study suggested ways to make science more inclusive and accessible. These suggestions include training STEM professionals in effective communication to engage with a diverse audience and translating scientific work into non-English languages to expand non-English sources in science (Márquez and Porras 2020). Mentored contributions from trainees to OERs such as Wikipedia offer opportunities to improve the quality and depth of openly available, domain-specific information and provides trainees with an authentic learning experience that has been shown to be beneficial for their learning (Forte and Bruckman 2006, Thomas *et al.* 2008). It has also been suggested that student initiatives are ideal for addressing systematic biases in Wikipedia (Ackerly and Michelitch 2022). One way to attract mentored contributions to OERs is through Wikipedia-editing competitions. Since 2012, the International Society for Computational Biology has run an annual Wikipedia competition for students (Bateman *et al.* 2013) and the value of leveraging this competition in a classroom setting has been discussed previously (Kilpatrick 2016, Kilpatrick *et al.* 2020).

In this study, we show how targeted educational activities and events can be used to narrow the knowledge gap between English and non-English OERs, using Spanish Wikipedia as a case study. We provide a comprehensive assessment of computational biology articles in Spanish Wikipedia, then analyse the improvements in article quality following an editing competition targeting these articles in Spanish Wikipedia. Here, we demonstrate that these contributions to Spanish Wikipedia narrow the knowledge gap to English Wikipedia. Finally, we provide some analysis to allow prioritization of article improvement initiatives, both in Spanish and in other non-English languages.

## 2 Materials and methods

Listing 1: SQL query used to retrieve Spanish language equivalents of English computational biology articles.

WITH t AS

(

SELECT

 
ll_lang, article.page_title AS en_title, ll_title AS es_title

FROM page talk

 
INNER JOIN page article ON talk.page_title = article.page_title

 
INNER JOIN categorylinks ON cl_from = talk.page_id

 
LEFT JOIN langlinks ON ll_from = article.page_id

WHERE

 
ll_lang = “es” AND

 
talk.page_namespace = 1 AND

 
article.page_namespace = 0 AND

 
cl_to IN (

 
 
”Top-importance_Computational_Biology_articles”,

 
 
”High-importance_Computational_Biology_articles”,

 
 
”Mid-importance_Computational_Biology_articles”,

 
 
”Low-importance_Computational_Biology_articles”,

 
 
”NA-importance_Computational_Biology_articles”,

 
 
”Unknown-importance_Computational_Biology_articles”

 
)

)

SELECT

 
ll_lang AS lang, en_title, es_title

FROM t

### 2.1 Defining computational biology articles

English Wikipedia articles relating to computational biology were defined as described previously (Kilpatrick *et al.* 2022). Briefly, these are articles tagged by Wikipedia editors as being within the scope of the Computational Biology workforce of WikiProject Molecular Biology. A list of tagged articles (https://wp1.openzim.org/#/project/Computational_Biology/articles) was retrieved via the WP1.0 bot, an automated tool which tracks Wikipedia article assessment data (Zheng *et al.* 2019).

Wikidata, the Wikimedia Foundation’s open knowledge base, maintains a list of interlanguage links, which link equivalent Wikipedia articles in different languages. For example, the English language article ‘Spain’ is linked via Wikidata to the Spanish article ‘España’. To generate a list of Spanish Wikipedia articles equivalent to our list of English computational biology articles, we used an SQL query (Code 1) to Wikimedia’s Quarry web service (https://meta.wikimedia.org/wiki/Research:Quarry) and downloaded the results in May 2022. As in similar analyses (e.g. Jemielniak *et al.* 2021), only those Wikipedia articles were compared that included encyclopedic content, removing three list pages from our analysis.

### 2.2 Article content assessment

Article importance, or relevance, for each WikiProject was rated by Wikipedia editors, on a four-point scale increasing through Low, Mid, High and Top importance (Kilpatrick *et al.* 2022). In this study, the importance ratings for the Computational Biology taskforce of WikiProject Molecular Biology was used, assuming that an article’s importance is independent of language.

**Figure 1.**
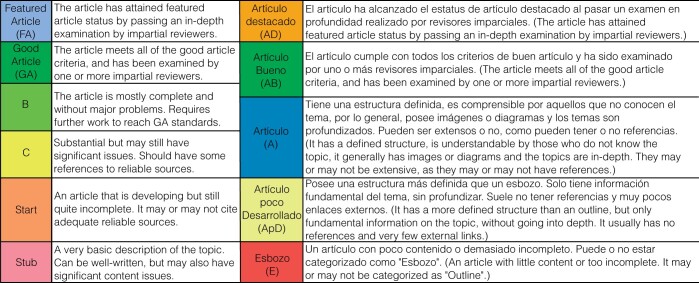
Article quality assessment criteria for English Wikipedia (left) and Spanish WikiProject Molecular Biology (right). More detailed criteria may be found at https://en.wikipedia.org/wiki/Wikipedia:Content_assessment and https://es.wikipedia.org/wiki/Wikiproyecto:Biolog\%C3\%ADa_celular_y_molecular, respectively. English Wikipedia Good Article criteria may be found at https://en.wikipedia.org/wiki/Wikipedia:Good_article_criteria.

English Wikipedia articles are rated for quality by Wikipedia editors according to defined quality criteria (Fig. 1); an article’s rating is accessible via its talk page. Articles of increasing quality may be rated from Stub (lowest quality) to the Good Article (GA) and Featured Article (FA) classes. To reach GA or FA class, articles must pass an internal peer review process; these classes are relatively rare, representing 0.5% and 0.1% of all Wikipedia articles, respectively.

Spanish Wikipedia does not have similar site-wide quality assessment criteria. However, WikiProjects may define their own article quality classes. In our assessment of Spanish Wikipedia computational biology articles, we used the ratings suggested by Spanish WikiProject Cellular and Molecular Biology (*Wikiproyecto Biología celular y molecular*). While these do not map exactly to English Wikipedia quality ratings, there is a similar progression from *Esbozo* (E; ‘outline’) to *Artículo destacado* (AD; ‘featured article’) classes (Fig. 1).

Articles were rated by final year undergraduate students in the Technologies for Information in Science program at UNAM Morelia, Mexico, all of whom had chosen biology as their terminal area and were writing their thesis in computational biology. Following an introductory workshop by Wikimedia-México where students were taught Wikipedia basics, articles were rated based on the *Wikiproyecto Biología celular y molecular* assessment criteria.

### 2.3 Spanish Wikipedia article improvement

Spanish Wikipedia articles were edited during the ISCB-LA SoIBio BioNetMx Wikipedia Competition (https://en.wikipedia.org/wiki/WP:ISCB-LA-2022). This competition, modelled on the ISCB Wikipedia Competition, was jointly organized by the ISCB Latin America 2022 conference (ISCB-LA; https://www.iscb.org/la2022), the Iberoamerican Society of Bioinformatics (SoIBio), and the Bioinformatics Network Mexico (BioNetMX). The competition was targeted at native Spanish and Portuguese speakers (students of any level and postdocs) to improve Spanish and Portuguese Wikipedia coverage of any topic relating to the ISCB’s Bioinformatics Core Competencies (v3.0) (Welch *et al.* 2014, 2016, Mulder *et al.* 2018). However, ultimately only Spanish language articles were entered into the competition. The competition ran between May and September 2022, with first, second, and third prizes offered for the best article improvements. A total of 25 student entrants with interest in bioinformatics, genomics and biomedicine claimed 25 articles to edit during the competition period, either individually or in groups. Award winners were subsequently invited to continue editing their chosen articles and submit them as entries to the international ISCB Student Wikipedia Competition. Following the editing periods, edited articles were rerated for quality as outlined above. Original page sizes and the amount of information added to each article, both in bytes, were extracted manually from article history pages, which record all previous edits to an article.

### 2.4 Data analysis

**Figure 2.**
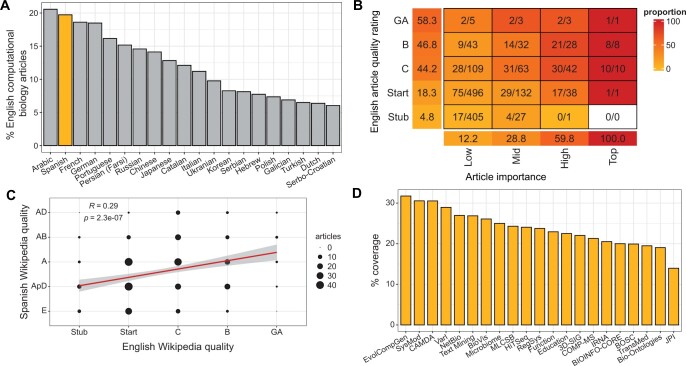
Quantitative assessment of computational biology articles in Spanish Wikipedia. (A) Barplot of non-English Wikipedias, ordered by the number of articles relating to computational biology (top 20 shown, highlighting Spanish Wikipedia). (B) Heatmap illustrating the proportion of English computational biology articles which have Spanish equivalents, for each combination of quality and importance classes. (C) Correlation of Spanish computational biology article quality with that of the equivalent article in English Wikipedia. (D) Barplot of ISCB COSIs, ordered by their coverage in Spanish Wikipedia.

**Figure 3.**
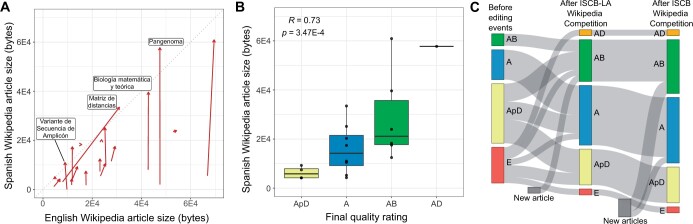
Targeted Spanish language activities narrow the knowledge gap. (A) Arrowplot showing the change in English Wikipedia (*x*-axis) and Spanish Wikipedia (*y*-axis) article sizes before and after the ISCB-LA SoIBio BioNetMx Wikipedia Competition, for articles modified as part of the competition. Winning articles are highlighted (see Table 1). The diagonal line represents equal article sizes in English and Spanish. (B) Article size vs quality rating for ISCB-LA SoIBio BioNetMx Wikipedia Competition entries, following the competition. (C) Sankey plot of article quality, for articles entered into the ISCB-LA SoIBio BioNetMx Wikipedia Competition and the ISCB Student Wikipedia Competition. Article quality is indicated at three timepoints: before and after the ISCB-LA SoIBio BioNetMx Wikipedia Competition, and after the ISCB Student Wikipedia Competition.

Data were imported into R (v.4.2.3) using the readxl tidyverse package (v.1.4.3) (https://www.tidyverse.org/). Article quality ratings for both English and Spanish articles were mapped to integer values (Stub = 1, Start = 2, etc.). The trend line and confidence intervals in Fig. 2C were computed using a generalized linear model using the stats R package. Correlations and significance values were computed using Spearman’s rank correlation coefficient (ρ). Heatmaps were created using the ComplexHeatmap Bioconductor package (Gu *et al.* 2016). The Sankey diagram in Fig. 3C was created with the networkD3 (v.0.4) R package (http://christophergandrud.github.io/networkD3/) Scripts for data analysis are available at: https://github.com/ISCBWikiTeam/spanish.

For computational biology domain-specific analysis, we make use of the COSI-Article matrix (v1) (Kilpatrick *et al.* 2022, Zenodo, doi: 10.5281/zenodo.5814765). This is a publicly-available dataset linking ISCB Communities of Special Interest (self-organizing groups of researchers focused on specific areas of computational biology) to computational biology articles from English Wikipedia.

The normalized article quality score is computed as in Jemielniak *et al.* (2021). Briefly, this is:

$$Q=\frac{N_{\text{quality}}}{\sqrt{N_{\text{total}}}},$$

where $N_{\text{quality}}$ is defined as the number of ‘quality articles’ within a WikiProject or taskforce (the sum of the articles in the peer-reviewed GA and FA classes) and $N_{\text{total}}$ is the total number of articles within that WikiProject or taskforce. Since there are no peer-reviewed Spanish Wikipedia articles, we use the equivalent AB and AD classes to compute a notional normalized article quality score.

The expectation ratio for a language’s Wikipedia is computed as:

$$\frac{N_{\text{compbio}}/N_{\text{total}}}{N_{\text{en}\_\text{compbio}}/N_{\text{en}\_\text{total}}},$$

where $N_{\text{compbio}}$ is the number of computational biology articles in a given language’s Wikipedia, $N_{\text{total}}$ is the total number of articles in that language’s Wikipedia, and $N_{\text{en}\_\text{compbio}}$ and $N_{\text{en}\_\text{total}}$ are the values for English Wikipedia. Higher expectation ratios indicate that a given language has greater computational biology coverage than may be expected, given the size of that language’s Wikipedia. The value for English is 1, by definition.

## 3 Results

### 3.1 Quantitative assessment of computational biology articles in Spanish Wikipedia

Our analysis of data returned from a Quarry SQL query indicates that 47.1% of English Wikipedia computational biology articles have no equivalent articles in non-English Wikipedias. Arabic Wikipedia has the highest proportion of shared articles (20.6%), followed by Spanish (19.7%) and French (18.6%) Wikipedias (Fig. 2A). There is a long tail of non-English languages with minimal computational biology presence; 30 languages only have a single article relating to computational biology.

The result of our SQL query identified 301 Spanish Wikipedia articles relating to computational biology. We found that the set of Top importance computational biology articles in English Wikipedia have equivalent articles in Spanish (Fig. 2B). As article importance decreases, an increasingly small proportion of articles have equivalents (High, 59.8%; Mid, 28.8%; Low, 12.2%). As previously noted, there were no English Wikipedia articles in the highest peer-reviewed FA class (Kilpatrick *et al.* 2022). Of the 12 English Wikipedia articles in the GA class, 7 (58.3%) have Spanish equivalents. As with article importance, we found an increasingly small proportion of articles with Spanish language equivalents with decreasing article quality (B, 46.8%; C, 44.2%; Start, 18.3%; Stub, 4.8%).

Results of the hackathon provide us with comparative indices for the quality of computational biology articles in Spanish and English Wikipedia. We identified a significant correlation between the quality ratings generated through our hackathon, and those of articles in English Wikipedia ($\rho =0.29,P=2.26\;\times \;10^{-7}$) (Fig. 2C). The most common quality rating for Spanish Wikipedia articles was A, (*Artículo*; article), representing 32.9% of articles. These were substantial articles with a defined structure and understandable by those unfamiliar with the topic. However, it is important to note that given the quality assessment criteria, the articles may not be extensive, or have sufficient references. 16.3% of articles were rated the lowest E (*Esbozo*, outline) class, a smaller proportion compared to the 29.9% of Stub class computational biology articles in English Wikipedia overall (Kilpatrick *et al.* 2022), but larger than the proportion of articles with Spanish language equivalents which are rated Stub class in English Wikipedia (7.0%). A total of 19.3% were rated as AB or AD, signifying articles of high quality that would be candidates for Wikipedia’s peer review process. Direct comparisons with English Wikipedia’s peer-reviewed GA class are likely to be complicated by the number of B class articles which are of GA standard but have not been peer-reviewed. However, we note that English equivalents of four Spanish articles rated here as AB or AD have already passed GA review.

To further analyse Spanish computational biology articles at a domain level, the data from the current study was combined with data from the COSI-Article matrix (Kilpatrick *et al.* 2022). This dataset links English Wikipedia articles to relevant ISCB Communities of Special Interest (COSIs), allowing analysis of the subfields of computational biology that are underrepresented in Spanish Wikipedia. Here, we identified that the Evolution & Comparative Genomics (EvolCompGen) COSI has the highest Spanish-language coverage (31.7%), while the Junior Principal Investigators (JPI) COSI was an unusually poorly-covered outlier (14.0%) (Fig. 2D). The JPI COSI also has the fewest relevant English language articles (*n *=* *43), many of which are biographies of computational biology researchers; we suggest that this explains the low coverage. Further analysis revealed no wider correlation between the number of English language articles relevant to a COSI and its coverage in Spanish Wikipedia (ρ=0.01,P=.96).

### 3.2 Targeted Spanish language activities narrow the knowledge gap

To assess the improvement in Spanish Wikipedia article quality following a targeted editing activity, we compared article size before and after the ISCB-LA SoIBio BioNetMx Wikipedia Competition (see Section 2). We also rerated the quality of articles entered into the competition. Twenty-five Spanish Wikipedia articles were entered into the competition; 19 of these were edited by participants within the 4.5 month competition period. Articles were edited by entrants a median of 7 times (range: 1 to 42). While some edits removed material from Wikipedia, a median of 5,222 bytes of information (range: −3–55 720) was added to each edited article, representing a median increase of 131% in article size (range: −0.02% to 2745%). Over the same period, the equivalent English Wikipedia articles had a median increase of 0.2% in article size (range: −8.82% to 289%). There is therefore an overall trend of Spanish language articles becoming closer in article size to their English language counterparts (Fig. 3A), although we note that academic writing in Spanish has been characterized as being less concise than that in English (Cuenca 2003).

While most entries edited existing Spanish Wikipedia articles, the article *Variante de Secuencia de Amplicón* (Amplicon sequence variant) was new: a translation of the equivalent English article. Interestingly, this article had not previously been tagged as relevant to the Computational Biology taskforce of WikiProject Molecular Biology. Article quality was rerated following the competition period, using the same assessment criteria. We computed an increase in the notional normalized article quality score (see Methods) for the 19 edited articles as a result of the edits made during the competition, from Q=0.69 to Q=1.61. We compute a similar increase in article quality for all articles entered into the competition (Q=0.60 to Q=1.40) and all Spanish language computational biology articles (Q=3.34 to Q=3.57). While page size is not a complete indicator of article quality, we found article size to be positively and significantly correlated with article quality ($\rho =0.73,P=3.47\times 10^{-4}$) (Fig. 3B).

Competition entries were judged based on factors including the clarity of writing, depth of knowledge of the subject area, pertinence to computational biology and use of relevant figures to illustrate the text. The winning articles are summarized in Table 1. Award winners were subsequently invited to continue editing their chosen articles and submit them to the international ISCB Student Wikipedia Competition. Two articles, *Biología matemática y teórica* and *Variante de Secuencia de Amplicón* were entered into the international competition. Further, two newly created Spanish language articles were entered into the international competition: *Índice Chao1* (Chao1 index) and *Tecnologías de transcriptómica* (Transcriptomics technologies). *Índice Chao1* was an article without an equivalent article in English Wikipedia. *Tecnologías de transcriptómica* was a translation of the equivalent English Wikipedia article, which had been developed and published previously (Lowe *et al.* 2017) through the *PLOS Computational Biology* Topic Pages initiative (Wodak *et al.* 2012). Together, these two new pages added 172 909 bytes of information to Spanish Wikipedia. Following the competition, their quality was rated A and AB, respectively. A final Spanish language entry, *Ecuaciones de Lotka-Volterra* (Lotka–Volterra equations), had an equivalent English Wikipedia article which had also not been previously tagged as relevant to the Computational Biology workforce of WikiProject Molecular Biology, despite dating from 2004.

**Table 1.** Winning articles in the ISCB-LA SoIBio BioNetMx Wikipedia Competition 2022.

Award	Article title (Spanish)	Article title (English)	Article importance	English article quality	Spanish article quality (before)	Spanish article quality (after)	Bytes added	Page size change (%)
1st	*Pangenoma*	Pan-genome	Mid	C	E	AD	55 720	2745
=2nd	*Matriz de distancias*	Distance matrix	High	Start	ApD	A	30 379	979
=2nd	*Variante de Secuencia*	Amplicon sequence	–	–	–	A	10 996	
	*de Amplicón*	variant						
3rd	*Biología matemática*	Mathematical and	Top	C	ApD	AB	31 592	393
	*y teórica*	theoretical biology						

For each article, its importance and quality in English Wikipedia, Spanish Wikipedia quality ratings before and after the competition, and page size metrics are provided. *Variante de Secuencia de Amplicón* was created during the competition; the Amplicon sequence variant article was not previously tagged by Wikipedia editors as being within the scope of the Computational Biology workforce of WikiProject Molecular Biology.

Overall, both targeted editing events quantifiably reduced the knowledge gap between Spanish and English Wikipedias, by expanding and improving existing Spanish articles and creating new Spanish articles of high quality (Fig. 3C).

### 3.3 Prioritization of initiatives in Spanish, English, and other languages

Analysis of our data also allows data-driven prioritization of initiatives to close the knowledge gap between Spanish and English Wikipedias, and improve computational biology coverage in other non-English Wikipedias. While most of the 20 Top importance computational biology articles are of comparable quality between English and Spanish Wikipedias, five articles (25%) have Spanish Wikipedia quality markedly lower than that in English Wikipedia: Biostatistics, Systems biology, Microarray, Computational Biology and RNA-seq. The Spanish equivalents of the latter three articles are all in the *Esbozo* quality class and should be prioritized for improvement. We find a greater knowledge gap in High importance articles, where 40.2% of English articles have no equivalent Spanish article. These include 7 articles (5.7%) rated B for quality, which should be prioritized for English to Spanish translation.

Four English Wikipedia articles in the GA quality class (33%) have no equivalent Spanish article: Circular permutation in proteins, Erez Lieberman Aiden, European Nucleotide Archive and Intelligent Systems for Molecular Biology. Again, these articles would be ideal candidates for translation, similarly to the translation of Transcriptomics technologies described above.

As a result of edits made during the ISCB-LA SoIBio BioNetMx Wikipedia Competition, six of the 19 Spanish articles edited (*Matriz de distancias*, *Nivel de calidad Phred* [Phred quality score], *Pangenoma*, *¿Qué es la vida?* [What is Life?], *Selección por torneos* [Tournament selection] and *Variante de Secuencia de Amplicón*) are now larger than their English equivalents. Their page sizes are median 18.8% larger in Spanish and suggest investigating if information may be transferred to the equivalent English Wikipedia articles.

**Figure 4.**
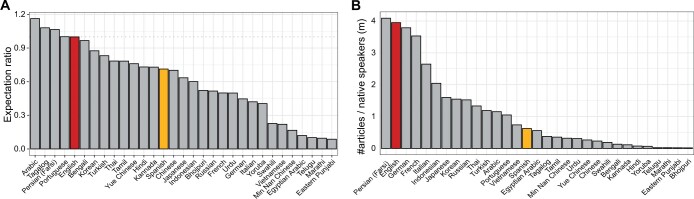
Prioritization of initiatives in Spanish, English, and other languages. (A) Barplot showing expectation ratio of computational biology articles given Wikipedia size. English and Spanish Wikipedias are highlighted. (B) Barplot showing number of computational biology articles, normalized by millions of native speakers.

While computational biology coverage in all non-English Wikipedias is significantly lower than that in English Wikipedia (Fig. 2A), combining our Quarry results with Wikimedia data on non-English Wikipedias and data for number of speakers (Eberhard *et al.* 2023) allows us to suggest which languages beyond Spanish should be prioritized in similar initiatives. By computing an expectation ratio (see Methods), we find that Arabic, Tagalog, Persian and Portuguese Wikipedias have higher than expected computational biology coverage, given the size of their Wikipedias (Fig. 4A). Many widely-spoken languages, such as Hindi, have fewer than expected articles relating to computational biology, even when normalizing to the smaller number of articles in that language’s Wikipedia. Normalizing the number of computational biology articles by the number of native speakers (Fig. 4B) highlights the lack of coverage given the number of Spanish speakers as we have discussed, but also that of languages such as Hindi. Chinese Wikipedia is a special case, as access to this has been restricted in mainland China since 2015. Interestingly, Arabic, Tagalog, and Portuguese score similarly to Spanish when normalizing by the number of native speakers, suggesting that although computational biology has higher coverage than expected in these languages given the Wikipedia size, initiatives to improve coverage in these languages should be prioritized, based on the number of native speakers.

## 4 Discussion

Despite the recent rapid growth in non-English Wikipedias, there remains significant knowledge gaps between these resources and English Wikipedia, even for widely spoken languages (Roy *et al.* 2021). We illustrate this here, with non-English Wikipedias covering barely 20% of computational biology content at best, and mostly in substantially less detail. Results from the current study support the notion that targeted editing events carry the potential to quantifiably reduce knowledge gap between Spanish and English Wikipedias by adding information to Spanish computational biology articles. While we see relatively little change in the English equivalent articles, we expect these articles to be more stable, since they are more complete at the beginning of the competition period.

Language barriers for non-English speakers in computational biology remain high, and non-English primary sources are more difficult to find, since publishing in English-language journals is more desirable for scientists due to the prestige associated with higher impact and increased exposure to disseminated research (Di Bitetti and Ferreras 2016). While there may be cultural or sociolinguistic contexts leading to some communities preferring to work in English rather than their native language, we maintain that reducing the knowledge gap between English and non-English languages remains important in improving computational biology OERs in a pedagogical setting.

There is an inevitable subjectivity in article quality ratings, even if rating criteria are well-defined (Kilpatrick *et al.* 2022). This may be reduced by having multiple volunteers rate article quality. Further, there have been few wide-scale efforts to assess information quality on Spanish Wikipedia (Ferretti *et al.* 2018), leading us to use the quality assessment criteria of *Wikiproyecto Biología celular y molecular* in this study. However, despite these limitations, we do observe a correlation in quality ratings between English and Spanish computational biology articles. One important follow-up task would be to submit the highest rated Spanish language articles to Spanish Wikipedia’s peer-review process; creation of a dedicated computational biology workforce within *Wikiproyecto Biología celular y molecular* would advance this goal. There is a further assumption that all English Wikipedia articles relating to computational biology have been tagged as such. However, during this study we found untagged but relevant articles, such as Amplicon sequence variant and Lotka–Volterra equations. If relevancy were defined based on ISCB core competencies, we expect that the number of relevant articles would be greatly increased. Systematically identifying such untagged but relevant articles, potentially via machine learning, would be an ideal hackathon activity.

Machine translation is a potential tool to increase the number of computational biology articles in Spanish Wikipedia. Indeed, a single translation bot, ‘Lsjbot’, is responsible for more than 6 million articles (99.6%) in the Cebuano (a language of the southern Philippines) edition of Wikipedia and more than 1 million in Swedish Wikipedia (Alshahrani *et al.* 2023). However, despite recent improvements, machine translation may still be limited by poor direct translation or shallow template-based translation, illustrating the continuing relevance of human translation efforts. This may be especially complicated in a technical field with many neologisms, such as computational biology, where direct translations do not exist.

Human translation is complicated by translation often not being made by the original author of an article. Indeed, the collaborative nature of Wikipedia means that articles are often written by several authors, and could be translated by several authors, each with their own interpretation of the original text, affecting translation quality. One solution may be to utilize multilingual members of the ISCB Student Council’s Regional Student Groups and ISCB affiliate groups, which operate worldwide. Only a quarter of multilingual Wikipedia editors edit the same articles in multiple languages (Hale 2014). Increasing this fraction would assist in closing the knowledge gap; here, we highlight the success of dual-language entries to previous editions of the ISCB Student Wikipedia competition (for example, in English and Chinese (O’Neill *et al.* 2017) and further encourage multilingual students participating in English to also submit entries in their native language. Efforts to gamify the translation process may also increase community participation and improve translation quality.

Large language models (LLMs) trained in multiple languages have recently been proposed to aid in bridging language barriers. However, LLMs are predominantly trained in English due to insufficient text data in other languages. For instance, GPT3’s training data consists of >90% English text by word count (data from Wikipedia made up 3% of the training mix) (Brown *et al.* 2020). Relying solely on one language for training generative AI can lead to disparities in user experiences across different languages, such as understanding and representing cultural nuances (Havaldar *et al.* 2023), common sense reasoning, question answering, or translation capability between languages. Encouraging students to contribute to OERs in their native languages may also lead to having more available resources to train LLMs. For instance, analysis of eight non-English Wikipedias has recently shown that almost 50% of articles in these Wikipedias have no English equivalent (Roy *et al.* 2021). Here, the creation of the *Índice Chao1* article highlights there may be further Spanish articles relevant to computational biology but with no English equivalent.

The wider knowledge gap between English and languages other than Spanish indicates a need to rapidly upscale translation efforts. Two of the current authors have used editing of Wikipedia articles in their teaching, encouraging students to work on translating articles as appropriate. We suggest that this approach is taken up more widely and have published guides for educators to assist with this (Kilpatrick *et al.* 2020). Encouraging ISCB COSIs to run domain-specific editathons (with Wikimedia support) may also help to upscale article creation and translation, reducing the knowledge gap.

## 5 Conclusion

Wikipedia is the most widely-used OER in computational biology. Beyond a static resource, mentored Wikipedia editing in a pedagogical setting is beneficial for learners, leading them to describe computational biology concepts in their own words. Creating new articles on computational biology topics also increases their coverage in OERs; however, there is an increasing knowledge gap between English and other languages. Here, we quantify the extent of the knowledge gap then, using Spanish Wikipedia as a case study, demonstrate that targeted educational activities can help reduce this knowledge gap. Finally, we discuss ways to prioritize and encourage the creation of computational biology resources in non-English languages. Such initiatives will increase equity, diversity and inclusion in computational biology and could equally be used in other scientific fields, with the impact of this felt beyond academia.

## Data Availability

The data underlying this article are available in GitHub and can be accessed at https://github.com/ISCBWikiTeam/spanish.
